# A Microfluidic Device Integrating a Glucose Sensor and Calibration Function for Cell-Based Assays

**DOI:** 10.3390/bios15050307

**Published:** 2025-05-11

**Authors:** Laner Chen, Kenta Shinha, Hiroko Nakamura, Kikuo Komori, Hiroshi Kimura

**Affiliations:** 1 Department of Mechanical Engineering, School of Engineering, Tokai University, Kitakaname, Hiratsuka 259-1292, Kanagawa, Japan; 2 Micro/Nano Technology Center, Tokai University, Kitakaname, Hiratsuka 259-1292, Kanagawa, Japan; 3 Department of Biotechnology and Chemistry, Faculty of Engineering, Kindai University, Takaya-Umenobe, Higashi-Hiroshima 739-2116, Hiroshima, Japan

**Keywords:** microfluidic system, microphysiological system (MPS), biosensor, glucose oxidase, calibration, cell-based assay, paraquat

## Abstract

Microphysiological systems (MPS) incorporating microfluidic technologies offer improved physiological relevance and real-time analysis for cell-based assays, but often lack non-invasive monitoring capabilities. Addressing this gap, we developed a microfluidic cell-based assay platform integrating an electrochemical biosensor for real-time, non-invasive monitoring of kinetic cell status through glucose consumption. The platform addresses the critical limitations of traditional cell assays, which typically rely on invasive, discontinuous methods. By combining enzyme-modified platinum electrodes within a microfluidic device, our biosensor can quantify dynamic changes in glucose concentration resulting from cellular metabolism. We have integrated a calibration function that corrects sensor drift, ensuring accurate and prolonged short-term measurement stability. In the validation experiments, the system successfully monitored glucose levels continuously for 20 h, demonstrating robust sensor performance and reliable glucose concentration predictions. Furthermore, in the cell toxicity assays using HepG2 cells exposed to varying concentrations of paraquat, the platform detected changes in glucose consumption, effectively quantifying the cellular toxicity responses. This capability highlights the device’s potential for accurately assessing the dynamic physiological conditions of the cells. Overall, our integrated platform significantly enhances cell-based assays by enabling continuous, quantitative, and non-destructive analysis, positioning it as a valuable tool for future drug development and biomedical research.

## 1. Introduction

In recent years, the microphysiological system (MPS), which combines microfluidic device technology with cell culture and assay approaches, has been considered an ideal approach for cell analysis in drug development and research [1,2,3,4,5]. Compared with traditional cell research approaches, these platforms are recognized for their numerous advantages, including more physiologically relevant cell culture conditions and efficient cell assay methods. For example, perfusion of the culture medium in these platforms can contribute to the delivery of nutritional supplements and simulate shear stress loading on cells, thereby mimicking in vivo conditions [6,7]. However, cell status measurement in previous MPSs is still challenging because traditional methods, such as cell staining and imaging, inevitably cause irreversible damage to cells. The obtained optical or quantified data are discrete, allowing for the analysis of real-time changes in cell status. Thus, the non-invasive and real-time quantified cell status analysis system is expected to be advanced in MPSs.

There has been intensive research in medical diagnostics aimed at developing a reliable cell analysis platform that integrates biosensors to monitor the in vitro transient fluxes involved in cellular metabolism under exposure to medical materials. Monitoring changes in the levels of key substances, such as oxygen, glucose, and lactate, facilitates the quantification of cell growth and proliferation rates through the cell’s glucose metabolism [8,9,10,11]. Electrochemical biosensors, which utilize oxidation−reduction reactions and measure the response current, are effective electronic components and have been widely applied to analyze bioactive samples. In many previous studies, biological recognition element-casting electrodes, such as those using glucose oxidase (GOD), have been widely employed in electrochemical analysis under cell culture conditions due to their ability to detect certain substances [12,13,14]. In the section on electrode materials, platinum (Pt) is considered a better electrode material, contributing to a higher sensitivity, resolution, and longer lifetime, which improves the performance of existing enzyme-modified electrodes compared with gold (Au) [15]. Combination enzyme-modified electrodes, along with conventional reference and counter electrodes, are expected to serve as biosensors for monitoring changes in physiological conditions in cell-based experiments [16].

These cell-based analysis platforms, which integrate electrochemical biosensors into microfluidic devices, are expected to monitor in vitro dynamic cell state changes under in vivo conditions mimicking [17]. With advances in existing devices that integrate biosensors, microfluidic devices obtained by photolithographic and planar techniques can be utilized due to certain advantages, such as sample holding and microfluidic flow control. These advantages enable it to measure low sample volumes with a higher sensitivity and faster response times. Additionally, the flow rate control and injection solution control at these microfluidic devices enable real-time adjustment of diffusion layers, allowing for the adaptation of the sensor’s sensitivity and linear responses. However, electrochemical sensor drift resulting from a decrease in enzyme activity leads to an uncertain prediction of analyte levels, limiting the platform to short-term monitoring only.

Some previous studies have reported comparing the status of cells under toxic conditions with that under non-toxic conditions to determine the changes in cell kinetic status under toxic conditions [18]. Another study reported that the cells’ glucose consumption varied between upstream and downstream glucose concentrations [19]. However, more than one sensor’s measurement result was discussed in these studies, which led to uncertain predictions because of individual differences in sensors or cells. In another study, the discussion focused on one sensor and showed great potential for helping to understand cell kinetic status changes under different conditions [20]. Thus, online sensor calibration focusing on one single sensor is expected to improve the lifetime and measurement stability of enzyme-modified biosensors in long-term monitoring.

In this study, we report on a cell-based assay platform featuring an integrated electrochemical glucose sensor designed to monitor kinetic changes in cell status during toxicity experiments. For certain predictions of glucose concentration and standalone cell analysis, the calibration function was integrated into the microfluidic device for recalibration of the biosensor. We evaluated the biosensor’s basic measurement function and the microfluidic device’s calibration function in a prolonged short-term measurement experiment. Then, we monitored the cell kinetic status through glucose consumption during a cell-based toxicological experiment to assess the performance of the cell-based assay platform.

## 2. Materials and Methods

### 2.1. Cell-Based Assay Platform

**Figure 1:**
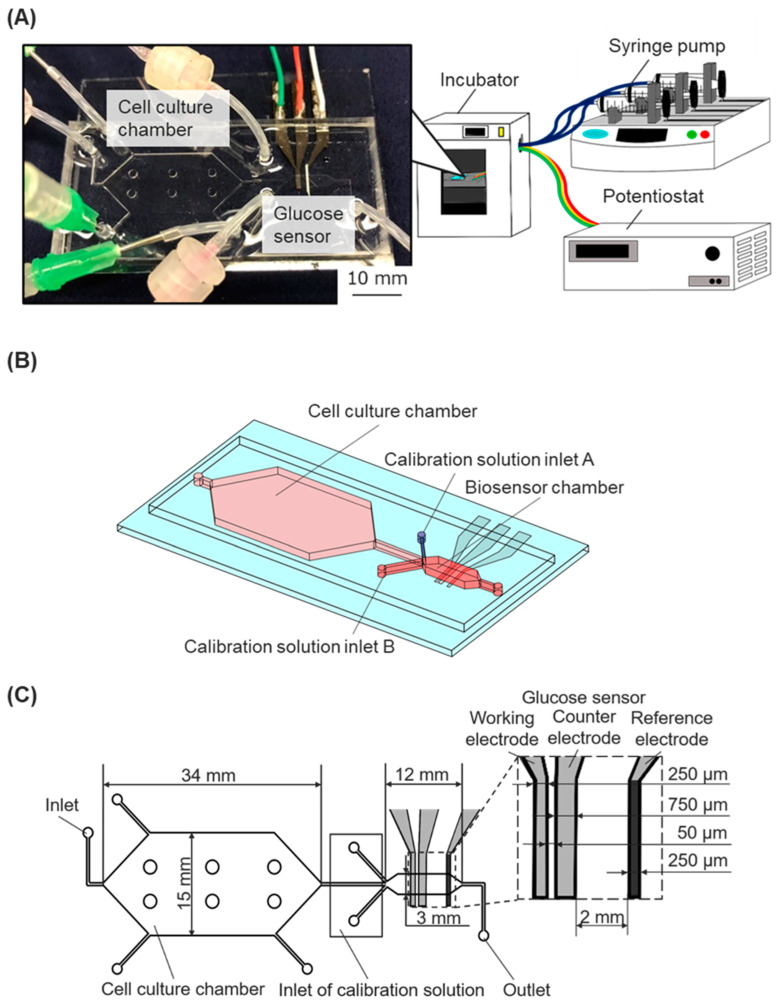
The cell-based assay platform consists of a microfluidic device with a glucose biosensor, a fluidic system, and a potentiostat. (**A**) Experimental setup: The microfluidic device, connected to syringe pumps and a potentiostat, was installed in an incubator. (**B**) Schematic image of the microfluidic device. (**C**) The geometry of the microfluidic device with the integrated glucose sensor.

We developed a cell-based assay platform that incorporates a microfluidic device with an enzyme-modified biosensor connected to syringe pumps and a potentiostat in order to monitor the dynamic state of cells (Figure 1A). The microfluidic device was fabricated by assembling a dimethylpolysiloxane (PDMS) chip with microchannel structures and a glass substrate with electrodes for an enzyme-modified biosensor.

### 2.2. PDMS Chip

The microfluidic chip had a cell culture chamber, a biosensor chamber, and microfluidic channels. The biosensor chamber was modulated downstream of the cell culture chamber (Figure 1B). The calibration solutions inlet A (for low glucose concentration solutions) and the calibration solutions inlet B (for high glucose concentration solutions) were situated near the main inlet of the sensor chamber. Three microfluidic channels of the inlets collapsed at the upper stream of the biosensor chamber. The cell culture chamber was designed with a height of 500 μm and a culture area of 530 mm². Six pillars (diameter 2 mm) were uniformly situated in the cell-culture chamber to prevent the attachment of the upper and bottom surfaces during the fabrication process. The biosensor chamber was designed with a height of 90 μm and a width of 3 mm (Figure 1C). The two chambers’ different heights and cross-sectional areas were designed to allow for less shear stress loading at the cell culture chamber, maintaining a constant flow rate [21].

The microfluidic chip was fabricated through conventional PDMS replica molding processes, and the negative pattern mold for the microfluidic chip was also created using a conventional photolithography process with negative photoresist SU-8 [22]. However, the SU-8 mold fabrication required two photolithography processes to build different thickness photoresist layers on a single Si wafer. A Si wafer cleaned with a piranha solution (a mixture of hydrogen peroxide (18084-00, KANTO KAGAKU, Tokyo, Japan) and sulfuric acid (37390-00, KANTO KAGAKU, Tokyo, Japan)) and coated with BHF was spin-coated at 1800 rpm with a layer of photoresist SU-8 2075 (Y111074, Nippon Chemical Industrial, Tokyo, Japan). The wafer was then exposed to UV light to obtain a template of the microchannel channels and sensor chamber with a thickness of 90 μm. The same photolithography process was conducted using SU-8 2100 (Y111075, Nippon Chemical Industrial, Tokyo, Japan) and a spin-coating speed of 800 rpm to obtain a mold of the culture chamber with a thickness of 500 μm. The position of the photo mask was adjusted using the microscope to align the attachment marks before UV exposure. The completed template was treated with CHF_3_ plasma (RIE-10NR, Samco, Kyoto, Japan) to deposit a fluorocarbon layer onto the surface of the SU-8 mold, ensuring the easy release of PDMS chips from the SU-8 mold.

The liquid-state PDMS (SILPOT184, Dow Toray, Tokyo, Japan) was poured into the SU-8 mold, and the bubbles were removed in a vacuum chamber. After 1 h and 30 min baking at 70 °C, the PDMS chip was hardened and ready to peel off from the SU-8 mold. Holes were made at all inlets and outlets using a biopsy punch (BP-40F, Kai Medical, Gifu, Japan), and these were connected to syringe pumps or medium reservoirs using silicone tubes (Figure 1A).

### 2.3. Enzyme-Modified Biosensor

The Pt electrodes on the glass structure were modified with glucose oxidase (GOD) to function as electrochemical biosensors for measuring the glucose concentration in the culture media. The sensor shapes and arrangement are shown in Figure 1C. Three-electrode electrochemical biosensors, comprising a working electrode, a reference electrode, and a counter electrode, were utilized. The working electrode (width, 250 μm) was positioned at the upper stream of the chamber, the counter electrode (width, 750 μm) was placed after the working electrode, and the reference electrode was positioned downstream of the chamber. The conventional photolithography process fabricated the thin-film electrodes on a glass substrate, using positive photoresist masking and aqua regia for wet etching. A 50.8 mm × 76.2 mm glass substrate coated with a thin platinum film (TOA Optical Technologies, Tokyo, Japan) was cleaned using ultrasonic cleaning and piranha solutions. It was then spin-coated with a layer of S1813G-positive photoresist (10336632, ROHM and HAAS Electronic Materials Korea Ltd., Seoul, Republic of Korea). The photoresist layer was exposed to UV light using a photo mask, and then the photoresist developer modified the layer to form electrode shapes. The glass substrate was wet etched using aqua regia (a mixture of hydrochloric acid (18078-00, Kanto Kagaku, Tokyo, Japan) and nitric acid (28161-00, KANTO KAGAKU, Tokyo, Japan)) to remove the Pt layer, thereby contributing to the shape of the Pt electrode. After wet etching, the glass structure was washed with phosphoric acid (32187-00, Kanto Kagaku, Tokyo, Japan) and acetone.

The reference and working electrodes were treated for electrochemical measurement of the glucose concentration. Initially, the reference electrode was fabricated using an Ag/AgCl ink (011464, BAS Inc., Osaka, Japan) to establish the standard potential for measurement. The GOD was coated onto the working electrode by chemical fixation. The enzyme solution was prepared by dissolving 2.5 mg GOD (G7141, Sigma-Aldrich, St. Louis, MO, USA) and 25 mg BSA (Bovine Serum Albumin, Sigma-Aldrich) as a cross-linking agent into a 250 μL PBS(-) solution (phosphate-buffered saline, D1408, Sigma-Aldrich, St. Louis, MO, USA) containing 0.02% Triton X-100 (020-81155, Kishida Chemical, Osaka, Japan), which serves as a surfactant. After the enzyme was dissolved, 15 μL of glutaraldehyde (17026-32, Kanto Kagaku, Tokyo, Japan) was added to the enzyme solution for chemical fixation. The mixture was then vortexed to ensure sufficient fusion. A 30 μL enzyme solution was dropped onto the surface of the working electrode using a thin PDMS film mask (250 μm thick PDMS film with a 4 mm diameter hole). A 30 s spin coating at 1500 rpm was then performed to remove excess enzyme solution. The sample was stored at 4 °C for 1 h. Then, 30 μL of 5wt% Nafion (527084, Sigma-Aldrich, St. Louis, MO, USA) was cast onto the surface of the sensing layer to protect it and prevent a decrease in enzyme activity due to plasma exposure during PDMS chip bonding, and was stored at 4 °C under stock conditions [23,24]. The PDMS chip was bonded onto the glass substrate with Pt electrodes via covalent bonds after oxygen plasma treatment using a plasma cleaner, resulting in a microfluidic device with an integrated glucose biosensor.

### 2.4. Electrochemical Measurement Principles

We first evaluated the basic performance of the Nafion/GOD-BSA/Pt electrode at a constant potential of ±0.4 V (vs. Ag/AgCl) in a 37 °C incubator and under a 9 μL/min perfusion condition to confirm the glucose concentration dependence of the biosensor. The glucose concentration range was set from 0 mM to 30 mM to maintain steady biosensor performance under normal culture conditions, encompassing the glucose concentration range between the no-glucose DMEM and high-glucose DMEM. All of the measurement processes were performed using an ALS/CH Instruments Electrochemical Analyzer Model ALS600B (BSA Inc., Osaka, Japan) linked to a personal computer in chronoamperometry mode. All of the measurements were collected at E = 0.40 V (±0.01 V) versus Ag/AgCl for glucose. The glucose concentration of the measurement solutions was adjusted to 0, 0.5, 1, 2, 4, 6, 8, 10, 15, 25, and 30 mM using the no-glucose DMEM culture medium (Dulbecco’s Modified Eagle’s medium, 11966-025, Thermo Fisher Scientific, Waltham, MA, USA) and the glucose powder (G8270, Sigma-Aldrich, St. Louis, MO, USA).

### 2.5. Constant Glucose Concentration Monitoring and Calibration Function

We confirmed the prolonged short-term performance of the biosensor under normal incubator conditions (37 °C, 20% CO_2_) and monitored the glucose concentration for 20 h to evaluate the stability of the biosensor under cell culture conditions. Low-glucose DMEM (Dulbecco’s Modified Eagle’s Medium, 12320–032, Thermo Fisher Scientific, Waltham, MA, USA) containing 5.6 mM glucose was used as an object solution. 

**Figure 2:**
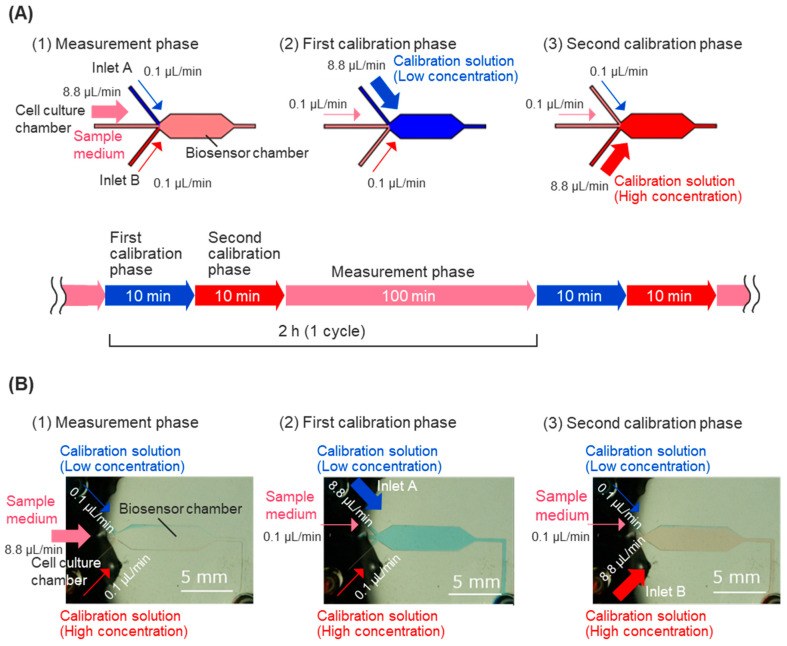
Automatic calibration procedure during prolonged short-term measurement. (**A**) During cell dynamics measurement, the sample culture medium comes from the cell culture chamber at a rate of 8.8 μL/min (1). During calibration, first, a calibration solution with a glucose concentration of 2 mM is introduced from calibration solution inlet A at a rate of 8.8 μL/min for 10 minutes (2). After that, a calibration solution with a glucose concentration of 8 mM is introduced at a rate of 8.8 μL/min from calibration solution inlet B for 10 minutes. Through these operations, the biosensor is calibrated every 2 h during prolonged short-term measurement. (**B**) Laminar flow of the solutions in the biosensor chamber during calibration using colored water. To prevent backflow, two ports other than the main port are also flowed at 0.1 μL/min in each phase.

For calibration of the biosensor, calibration solutions containing high and low concentrations of glucose were injected into the sensor chamber intermittently, allowing for the measurement of the response current’s decline over time. The linear response of the sample medium’s glucose concentration was measured, and calibration processes were executed every 2 h during the usual measurement. The calibration process consists of two calibration phases: the first calibration phase and the second calibration phase (Figure 2A). Calibration solutions were prepared using low-glucose DMEM containing 10% fetal bovine serum (FBS, 10270106, Thermo Fisher Scientific, Waltham, MA, USA), 25 mM HEPES, and varying glucose concentrations. In the first calibration phase, low-concentration glucose calibration solutions (containing 2 mM glucose) flowed into the biosensor chamber from inlet A for 10 min to obtain the peak value of the calibration curve. In the second calibration phase, high-concentration glucose calibration solutions (containing 8 mM glucose) flowed into the biosensor chamber from calibration solution inlet B for 10 min to obtain the foot value of the calibration curve. The fluidic control of the sample medium and calibration solutions was achieved automatically, using programming for syringe pumps to control the flow rate of each inlet (Figure 2A).

### 2.6. Cell Culture and Cell Toxicity Experiment

Human hepatocarcinoma liver cells (HepG2) were obtained from ATCC (Lot58210525, HB-8065, American Type Culture Collection, Manassas, VA, USA) and employed in the experiments, employing the culture medium of low-glucose DMEM containing 10% FBS and 1% antibiotics antimycotic solution (161-23181, FUJIFILM Wako Pure Chemical Corporation, Osaka, Japan). Before cell seeding, the cell-culture chamber was injected with Cellmatrix type I-P (Collagen Type I, dissolved in diluted hydrochloric acid, 634-00663, Nitta Gelatin, Osaka, Japan) and incubated for 1 h to coat the glass substrate with collagen. The chamber was then rinsed gently twice with the following culture medium. HepG2 cells were seeded into the cell culture chamber at an optimal seeding density of 2 × 10^5^ cells/cm^2^, and the cells were allowed to stand for 24 h, ensuring the cells were attached to the base and reached a steady state.

The HepG2 cells were cultured under fluidic conditions, and the glucose concentrations of the culture medium were monitored online using the biosensor in the microfluidic device. A culture medium was injected into the cell culture chamber to provide nutritional supplements to the cells, and then flowed through the biosensor chamber to measure the glucose concentration. A flow rate of 9.0 μL/min was applied during perfusion cell culture. 

A toxicity assay using paraquat (DSN3228, FUJIFILM Wako Pure Chemical Corporation, Osaka, Japan) was performed to evaluate the function of our system as a cell-based platform. Then, 0 mM, 5 mM, and 10 mM paraquat were dissolved in the following culture medium and applied in a cell toxicity experiment for 24 h. The glucose consumption (glucose concentration changes) was monitored during 24 h paraquat exposure as an index for predicting the cell’s kinetic status changes. The glucose concentration’s prolonged short-term measurement used the same protocol as the functional evaluation experiment (Figure 2A). 

### 2.7. Glucose Concentration Calculation

The glucose concentrations during prolonged short-term monitoring for evaluating calibration function and prolonged short-term monitoring in the cell toxicity experiments were calculated from the calibrated current values. The response current was calibrated using a current decline rate calculated from the difference between the current values in the calibration processes before and after the measurement phase.

In every calibration process (*n^th^*), the response current values (*I_low_* and *I_high_*) and the calibration solutions’ glucose concentrations (*C_low_* and *C_high_*) were used to calculate the sensitivity (*a_calibration(n)_*, Equation (1)) and the estimated base current value at a glucose concentration of 0 mM *(I*_0*(n)*_). Then, the sensitivity (*a_calibration(n)_*) and the estimated base current value *(I*_0*(n)*_) were used to establish Equation (2), which expresses the relationship between the current value (*I_calibration(n)_*) and the glucose concentration (*C*).

(1) $$a_{calibration(n)}=\frac{I_{high}-I_{low}}{C_{high}-C_{low}},$$

(2) $$I_{calibration(n)}=a_{calibration(n)}\times C+I_{0(n)}$$

The estimated standard current values (*I_standard_*) of the low-glucose medium during the calibration processes before (*n −* 1) and after (*n*) the measurement phase was calculated, based on Equation (2), for prediction of the current decline within the measurement phase. The current decline rate (*a_decline(n,n−_*_1*)*_) between two calibration processes (*n* and *n −* 1) was calculated using the difference between the estimated standard current values (*I_standard(n)_* and *I_standard(n−_*_1*)*_) and the time in one cycle period (*T* = 7.200 s) (Equation (3)).

(3) $$a_{decline(n)}=\frac{I_{standard(n)}-I_{standard(n-1)}}{T}$$

The current measured in the measurement phase (*I_t_*) was calibrated with the current decline rate (*a_decline(n,n−_*_1*)*_) using Equation (4), which consists of the calibrated current value (*I_estimated(t)_*), the response current value (*I_t_*), the time in one cycle period (*T*), and measured time in the cycle (*t*).

(4) $$I_{estimated(t)}=I_{t}-a_{decline(n,\;n-1)}\times (t-T)$$

Finally, the glucose concentration (*C_estimated(t)_*) in the sample medium was calculated from calibrated current values, using the sensitivity (*a_calibration(n)_*) and the estimated base current value (*I*_0*(n)*_) (Equation (5)).

(5) $$C_{estimated(t)}=\frac{I_{estimated(t)}-I_{0(n)}}{a_{calibration(n)}}$$

## 3. Results

### 3.1. Electrochemical Properties of Nafion/GOD-BSA/Pt Biosensor

The basic performance of the Nafion/GOD-BSA/Pt biosensor was evaluated using amperometry measurements in DMEM containing 0–30 mM at fluidic conditions. The GOD molecule is known to catalyze the oxidization of glucose with molecular oxygen to gluconolactone and to produce H_2_O_2_. Thus, the oxidation of H_2_O_2_ was carried out at the Pt electrode, contributing to electron transfer. The concentration of glucose is related to the rate of both oxidation reactions [25,26]. The possible reaction mechanisms are as follows.

(6) $$O_{2}+glucose\overset{GOD}{\to }Gluconolactone+H_{2}O_{2}$$

(7) $$H_{2}O_{2}\to {2H}^{+}+O_{2}+2e^{-}$$

**Figure 3:**
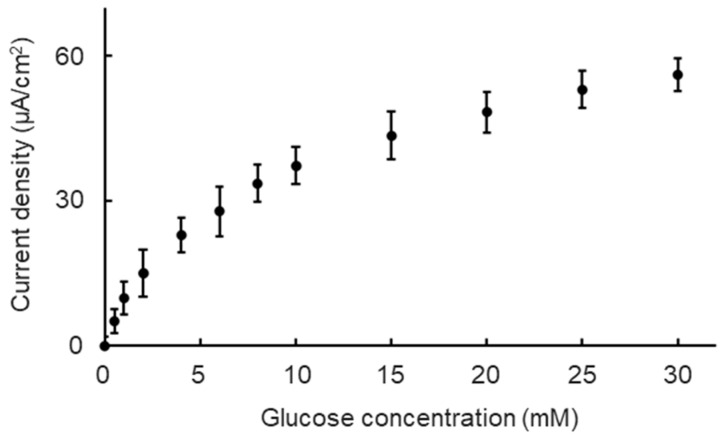
Biosensor outputting current density versus glucose concentration in DMEM (pH 7.4) at 37 °C. Values indicate mean ± standard deviation.

As shown in Figure 3, the anodic current was dependent on glucose concentrations at the fluidic condition. The current response increased significantly and then gradually increased as the glucose concentration increased. According to the results, the Michaelis−Menten kinetic mechanism was used to show the characteristics of the biosensor. The relationship between the current response and glucose concentration was expressed with the Lineweaver−Burk type equation (Equation (8)) [27], which consists of the current (I), the glucose concentration (C), the apparent Michaelis−Menten constant (Kmapp), and the maximum anodic current (Imax).

(8) $$\frac{1}{I}=\frac{K_{m}^{app}}{I_{max}}\left(\frac{1}{\left[C\right]}\right)+\frac{1}{I_{max}}$$

Based on the equation, I_max_ and K_m_^app^ were calculated to be approximately 44 μA/cm^2^ and 2.9 mM, indicating that the biosensor has a basic function to detect the glucose at different concentrations. However, the obtained K_m_^app^ value was lower than that for the free enzyme in the solutions (33 mM) [28]. This result indicates that structural distortion due to attaching enzymes onto the substrate caused retardation of the catalytic reaction rates, leading to a low value of K_m_^app^.

The glucose concentration range of 0 mM to 30 mM was divided into the low concentration range of 0 mM to 2 mM, the middle concentration range of 2 mM to 10 mM, and the high concentration range of 10 mM to 30 mM. Three ranges were divided to correspond to the glucose concentration of three types of universal culture medium: the no-glucose DMEM (0 mM glucose), the low-glucose DMEM (5.6 mM glucose), and the high-glucose DMEM (25 mM glucose). The sensitivities and resolutions of the biosensor at different ranges were quantified as a validation of the biosensor’s basic performance. The calculated sensitivities and resolutions of the biosensor at different glucose concentration ranges are shown in Table 1. The sensitivity decreased, and the resolution increased as the glucose concentration range increased. However, deviations in sensitivities and resolutions at every glucose concentration range did not show significant differences.

From the essential performance evaluation of the experimental results, the biosensor performed well in fluid conditions. This indicates that the perfusion conditions created steady and constant glucose supplements, leading to a steady response current that contributed to a higher sensitivity and detection ability (resolution). The glucose concentration’s variation after the medium passed through the cell culture chamber was calculated using the following equation (Equation (9)), which consisted of glucose concentration’s variation (*D_cells_*), the HepG2 cells’ glucose consumption rate (*GCR* = 4.1 × 10^−16^ mols^−1^cell^−1^) [29], the time for the culture medium to pass through the cell culture chamber (*t* = 1770 s), the total number of cells (*N* = 10^6^), and the volume of the cell culture chamber (*V_chamber_* = 265 μL).

(9) $$D_{cells}=\frac{GCRtN}{V_{chamber}}$$

Based on the equation, the glucose concentration’s variation was calculated at about 2900 μM. Thus, the essential resolution that securing the glucose concentration changes can be monitored must be below 2900 μM. Both biosensors’ resolutions at high, mid, and low glucose ranges were greater than the required resolutions. The performance of the biosensor met the requirements of our platform. 

### 3.2. Monitoring of Constant Glucose Concentration

First, the flow distributions in the biosensor chamber were evaluated to confirm the object solution complete reaction at the working electrode and to ensure the measurement’s accuracy in the calibration phases. The flow rate distribution of the main inlet and two calibration solutions inlets was adjusted within a total flow rate of 9.0 μL/min to control the distribution of solutions in the biosensor chamber. The changes in distribution for solutions were observed with a stereo microscope (SMZ25, Nikon, Tokyo, Japan), using red and blue pigment solutions. The solution distribution caused by a flow rate distribution of 8.8:0.1:0.1 μL/min is shown in Figure 2B. The object solutions nearly filled the sensor chamber and covered the enzyme casting area in the following flow rate distribution. 

**Figure 4:**
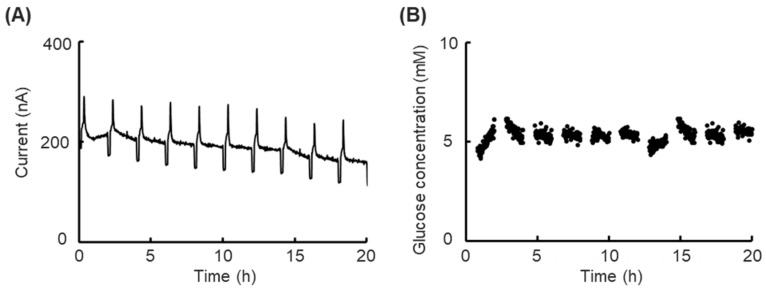
Results of prolonged short-term glucose concentration monitoring to evaluate the calibration function without cells. (**A**) Time course of the sensor response current in the culture medium with low-glucose DMEM containing 5.6 mM glucose at 37 °C with the calibration function. (**B**) Time course of the calculated glucose concentration using calibration data in the culture medium with low-glucose DMEM containing 5.6 mM glucose at 37 °C.

Then, the prolonged short-term monitoring experiment was executed. The response current during the prolonged short-term monitoring within 20 h is shown in Figure 4A, where it slightly declined at a constant 5.6 mM glucose concentration, and the clear response current peak and feet appeared at each calibration process after every 2 h of monitoring. Real-time glucose concentrations in every measurement phase were calculated using the following calculating method (Section 2.7); the result is shown in Figure 4B. Against a slightly lowering response current, the calculated real-time glucose concentration was maintained at nearly 5.6 mM, which conformed with the measurement results from the glucose analyzer (GP-9, Analox Instruments, London, UK).

The biosensor’s slight decline in response current can be considered the sensor’s drift due to decreasing enzyme activity. The calculated real-time glucose concentration maintained a consistent level, indicating the calibration function is valid for certain predictions of glucose concentration. In the enzyme-modified biosensors used in the cell-based studies reported to date, the biosensor’s drifts during the cell status monitoring were fixed by normalization with the control group [18]. However, the cell status difference between experimental groups will significantly influence the accuracy of cell drug toxicity experiments. In other studies, the drifts were fixed by comparing the difference in glucose concentration at the inlet or outlet of a cell culture chamber [19]. However, any single biosensor will easily interfere with the experimental results. In this study, all discussions focused on one single biosensor to avoid all external interference and inter-individual variabilities. The results showed the calibration function could eliminate the influence of biosensor drift during prolonged short-term monitoring and contributed to certain predictions of the objective’s concentration. The glucose concentration in monitoring was maintained within near 5.6 mM, and non-forecast changes could be individually discussed. Furthermore, the calibration processes executed at intervals between every measurement process and the lack of a requirement to remove the platform from the incubator contributed to the continuousness of measurement. Thus, our platform is readily available for glucose concentration prediction and advanced cell status monitoring.

### 3.3. Glucose Concentration Monitoring at Cell Toxicity Experiment

**Figure 5:**
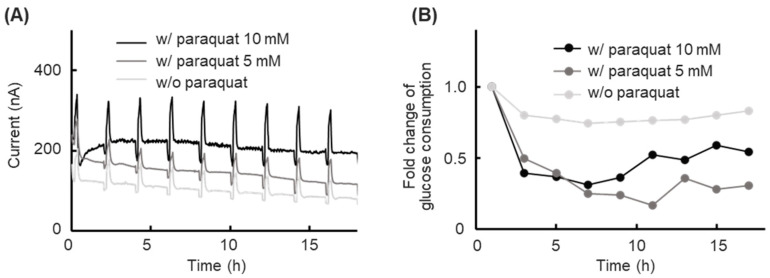
Results of prolonged short-term glucose concentration monitoring in a cell toxicity experiment. (**A**) Time course of the sensor response current for cells without paraquat, cells with 5 mM paraquat, and cells with 10 mM paraquat in a culture medium (low-glucose DMEM containing 5.6 mM glucose), with automatic calibration performed every 2 h. (**B**) Time course of the glucose consumption for cells without paraquat, cells with 5 mM paraquat, and cells with 10 mM paraquat in the culture medium, as determined by automatic calibration. Values indicate the moving average. The relative glucose consumption value was normalized with the initial glucose consumption value.

Paraquat, known as an international forbidden non-selective herbicide, leading to significant cell toxicity and DNA damage, was applied in a cell toxicity experiment [30,31,32]. The glucose concentration in the cell toxicity experiment was monitored to validate the usability of our platform at dynamic glucose concentration conditions. The response current during the cell toxicity experiments is shown in Figure 5A, the peaks of the response current from the calibration processes were observed every 2 h during the experiment. The response current level was clearly different depending on the paraquat dose, and a general downward drift phenomenon was observed. The calculated glucose consumption is shown in Figure 5B. The calculated glucose consumption was expressed as a moving average and normalized with the initial glucose consumption value. The group at culture conditions with 5 mM and 10 mM paraquat showed a significant decrease in glucose consumption within the first 7 h. After the glucose consumption decreased, the glucose consumption of both groups exposed to paraquat tended to stabilize. 

In a previous study on paraquat toxicity to HepG2 cells, it was reported that cell viability significantly decreased as the paraquat concentration increased [33]. However, the cell assay in this previous study was non-continuous, and they were unable to obtain more detailed information during paraquat exposure. In our study, the cell kinetic status changes were chronologically quantified evaluated by prolonged short-term monitoring of the glucose consumption. A decrease in glucose consumption, induced by paraquat’s cell toxicity, was observed on our platform. From the results, our platform showed the usability of cell-based analysis and we expect to apply it in more cell experiments and studies. All of the cell status analyses and discussions focused on data measured by a single biosensor, which were then compared under other conditions, made possible by a calibration function that enabled measurements to be taken at a single biosensor. Moreover, all of the experimental results proved our platform is more suitable for cell-based analysis in certain situations.

We conducted multiple independent experiments using different chips. As a result, we observed variability in the output current levels between chips. However, we confirmed that accurate glucose levels could be obtained by precisely calibrating each chip. As the sensors are currently handmade, it is technically challenging to eliminate inter-chip variability entirely. In the future, industrial-scale production would likely reduce such variability.

## 4. Conclusions

In this study, we presented a platform for the analysis of changes in the kinetic physiological conditions of cells by integrating an enzyme-casting electrochemical biosensor to obtain real-time glucose consumption changes and apply it to cell toxicity experiments. The cell kinetic status changes were realized as glucose consumption changes, which were obtained through a response current from the enzyme’s oxidation−reduction reactions. The calibration functions were achieved by spatiotemporally controlling the glucose concentration of the cell culture medium and detecting the decline in current to calibrate the biosensor’s drift. The prolonged short-term monitoring of the glucose level experiment demonstrated that glucose concentration could be accurately monitored over 20 h, providing a relative prediction of the glucose concentration, which was made possible by the calibration function. In the cell toxicity experiment, the toxicity of the paraquat was observed on our platform, demonstrating that monitoring more detailed changes in cell kinetic status is possible. The results of the experiments show that our platform is suitable for cell-based assays, and more medical development studies are expected to be applied. 

In future studies, glucose dehydrogenase (GDH), which does not consume oxygen and will not generate hydrogen peroxide, is considered a better glucose detection enzyme and is expected to be applied to improve the biosensor’s performance. Also, anti-biofouling coating materials are expected to be applied to extend the biosensor’s lifespan. The presented platform is expected to be further developed through longer-term evaluating experiments, so as to confirm its suitability for cell-based assays.

## Figures and Tables

**Table 1:** Results in biosensor’s sensitivity and resolution at culture medium glucose concentration ranges of 0–2 mM, 2–10 mM, and 10–30 mM. Values indicate mean ± standard deviation.

**Glucose Concentration Range (mM)**	**0** **–2**	**2** **–10**	**10** **–30**
Sensitivity (nA/mM)	50.8 ± 10.8	22.7 ± 7.4	7.0 ± 0.6
Resolution (μM)	6.8 ± 1.4	24.3 ± 5.0	43.7 ± 3.8

## Data Availability

The original contributions presented in this study are included in the article. Further inquiries can be directed to the corresponding author.

## References

[1] Dongeun H., Yu-suke T., Geraldine A.H., Hyun J.K., Donald E.I. (2012). Microengineered physiological biomimicry: Organs-on-chips. Lab A Chip.

[2] Kimura H., Sakai Y., Fujii T. (2018). Organ/body-on-a-chip based on microfluidic technology for drug discovery. Drug Metab. Pharmacokinet..

[3] Sung J.H., Srinivasan B., Esch M.B., McLamb W.T., Bernabini C., Shuler M.L., Hickman J.J. (2014). Using physiologically-based pharmacokinetic-guided “body-on-a-chip” systems to predict mammalian response to drug and chemical exposure. Exp. Biol. Med..

[4] Kimura H., Nishikawa M., Kutsuzawa N., Tokito F., Kobayashi T., Kurniawan D.A., Shioda H., Cao W., Shinha K., Nakamura H. (2025). Advancements in microphysiological systems: Exploring organoids and organ-on-a-chip technologies in drug development—Focus on pharmacokinetics related organs. Drug Metab. Pharmacokinet..

[5] Jun Y., Lee J.S., Choi S., Yang J.H., Sander M., Chung S., Lee S.H. (2019). In vivo–mimicking microfluidic perfusion culture of pancreatic islet spheroids. Sci. Adv..

[6] Ziółkowska K., Kwapiszewski R., Brzózka Z. (2011). Microfluidic devices as tools for mimicking the in vivo environment. New J. Chem..

[7] Huang H.C., Chang Y.J., Chen W.C., Tsai C.H., Chen Y.H., Chen Y.C. (2013). Enhancement of renal epithelial cell functions through microfluidic-based coculture with adipose-derived stem cells. Tissue Eng. Part A.

[8] Tahirbegi I.B., Ehgartner J., Sulzer P., Zieger S., Kasjanow A., Paradiso M., Strobl M., Bouwes D., Mayr T. (2017). Fast pesticide detection inside microfluidic device with integrated optical pH oxygen sensors and algal fluorescence. Biosens. Bioelectron..

[9] Koo K.M., Kim C.D., Ju F.N., Kim H., Kim C.H., Kim T.H. (2022). Recent advances in electrochemical biosensors for monitoring animal cell function and viability. Biosensors.

[10] Gonzalez-Guerrero M.J., Raboso-Gallego T., Fernandez-Sanchez C., de Marcos S., Lopez-Ruiz B. (2017). Paper-based microfluidic biofuel cell operating under glucose concentrations within physiological range. Biosens. Bioelectron..

[11] Kumar P., Nagarajan A., Uchil P.D. (2018). Analysis of cell viability by the lactate dehydrogenase assay. Cold Spring Harb. Protoc..

[12] Wong C.M., Wong K.H., Chen X.D. (2008). Glucose oxidase: Natural occurrence, function, properties and industrial applications. Appl. Microbiol. Biotechnol..

[13] Iogr A., Schuhmann W., Huber J. (2010). Enzyme electrodes to monitor glucose consumption of single cardiac myocytes in sub-nanoliter volumes. Biosens. Bioelectron..

[14] Bollella P., Katz E. (2020). Enzyme-based biosensors: Tackling electron transfer issues. Sensors.

[15] O’Neill D., Chang S.C., Lowry J.P., McNeil C.J. (2004). Comparisons of platinum, gold, palladium and glassy carbon as electrode materials in the design of biosensors for glutamate. Biosens. Bioelectron..

[16] Komori K., Komatsu Y., Nakane M., Sakai Y. (2021). Bioelectrochemical detection of histamine release from basophilic leukemia cell line based on histamine dehydrogenase-modified cup-stacked carbon nanofibers. Bioelectrochemistry.

[17] Sekhwama M., Mpofu K., Sudesh S., Mhlanga S. (2024). Integration of microfluidic chips with biosensors. Discov. Appl. Sci..

[18] Kimura H., Takeyama H., Komori K., Yamamoto T., Sakai Y., Fujii T. (2010). Microfluidic device with integrated glucose sensor for cell-based assay in toxicology. J. Robot. Mechatron..

[19] Kimura H., Shono Y., Pereira-Rodrigues N., Yamamoto T., Sakai Y., Fujii T. (2010). On-chip glucose sensor for online measurement of cell activities. IEEJ Trans. Sens. Micromach..

[20] Komori K., Usui M., Hatano K., Hori Y., Hirono K., Zhu D., Tokito F., Nishikawa M., Yasuyuki S., Kimura H. (2022). In vitro enzymatic electrochemical monitoring of glucose metabolism and production in rat primary hepatocytes on highly O_2_ permeable plates. Bioelectrochemistry.

[21] Tanaka Y., Yamato M., Okano T., Kitamori T., Sato K. (2006). Evaluation of effects of shear stress on hepatocytes by a microchip-based system. Meas. Sci. Technol..

[22] Hosokawa K., Fujii T., Endo I. (1999). Handling of picoliter liquid samples in a poly(dimethylsiloxane)-based microfluidic device. Anal. Chem..

[23] Fortier G., Vaillancourt M., Bélanger D. (1992). Evaluation of nafion as media for glucose oxidase immobilization for the development of an amperometric glucose biosensor. Electroanalysis.

[24] Rishpon J., Gottesfeld S., Campbell C., John D., Thomas A.Z. (1994). Amperometric glucose sensors based on glucose oxidase immobilized in Nafion. Electroanalysis.

[25] Bankar S.B., Bule M.V., Singhal R.S., Ananthanarayan L. (2009). Glucose oxidase—An overview. Biotechnol. Adv..

[26] Mandpe P., Prabhakar B., Gupta H., Shende P. (2020). Glucose oxidase-based biosensor for glucose detection from biological fluids. Sens. Rev..

[27] Kamin R.A., Wilson G.S. (1980). Rotating ring-disk enzyme electrode for biocatalysis kinetic studies and characterization of the immobilized enzyme layer. Anal. Chem..

[28] Swoboda B.E.P., Massey V. (1965). Purification and properties of the glucose oxidase from Aspergillus niger. J. Biol. Chem..

[29] Shirakashi R., Yoshida T., Provin C., Sato K., Funamoto K., Takahashi M., Hibino T., Takiguchi T. (2007). Steady measurement of glucose metabolism of hepatocyte. Proceedings of the Heat Transfer Summer Conference.

[30] Teixeira H., Proença P., Alvarenga M., Oliveira M., Marques E.P. (2004). Vieira, D.N. Pesticide intoxications in the centre of Portugal: Three years analysis. Forensic Sci. Int..

[31] Urbančíková M., Korytár P. (1999). Cu-complex counteracts the effect of paraquat on polymerized actin. Toxicol. Vitr..

[32] Lee D.Y., Kang H.W., Kaneko S., Kwon Y.S., Muramatsu H. (2009). Direct monitoring of paraquat induced cell death using quartz crystal sensor. Thin Solid Film..

[33] Vrzal R., Zenata O., Doricakova A., Dvorak Z. (2015). Environmental pollutants parathion, paraquat and bisphenol A show distinct effects towards nuclear receptors-mediated induction of xenobiotics-metabolizing cytochromes P450 in human hepatocytes. Toxicol. Lett..

